# The value of multi-year sampling for detecting fine-scale population genetic structure in marine fishes: A case study of juvenile southern flounder

**DOI:** 10.1371/journal.pone.0348919

**Published:** 2026-09-22

**Authors:** Sydney P. Harned, Jamie L. Mankiewicz, Russell J. Borski, John Godwin, Martha O. Burford Reiskind

**Affiliations:** Department of Biological Sciences, North Carolina State University, Raleigh, North Carolina, United States of America; Central Marine Fisheries Research Institute, INDIA

## Abstract

Understanding population structure is critical for effective fisheries management in species with complex life histories and variable recruitment. Southern flounder (*Paralichthys lethostigma*) is a valuable flatfish species with declining populations in the Southeast United States. Improved management may depend on a better understanding of fine-scale and temporal population genetic structure in this region; however, such structure remains poorly characterized. To address our lack of understanding of the spatial and temporal population structure of this important species, we used double digest reduced-representation genome sequencing (ddRADSeq) on juveniles from estuaries in North Carolina and Texas between 2014 and 2023. We found significant genetic differentiation between the Gulf of Mexico and Atlantic populations, supporting the management of these regions as distinct stocks. By contrast, we detected significant variance in genetic structure within Texas and North Carolina populations that was not consistent across sampling years between estuaries in close proximity. The population genetic structure of southern flounder suggests significant, temporally variable genetic differences within estuarine locations that may result from variation in larval dispersal and recruitment patterns. Our findings highlight the value of integrating fine-scale, multi-year genetic data to capture temporal dynamics and avoid misleading conclusions based on single-year or broad-scale sampling.

## Introduction

The assessment of genetic structure and population connectivity is fundamental for understanding recruitment dynamics in marine species [1,2]. However, high dispersal potential and pelagic larval stages can make genetic population structure difficult to resolve [3,4]. While high dispersal often leads to high connectivity in marine systems, many marine species exhibit population structure despite this potential [5]. Advances in genomic sequencing now enable the analysis of tens of thousands of genomic markers, improving the detection of fine-scale population structure found in these dynamic marine systems [5]. These advances have improved our understanding of population structure across space, but to fully characterize population structure in a region requires replication in time as well. While pooling samples across years can provide a useful, time-averaged view of connectivity, this approach may obscure temporal variability driven by stochastic larval dispersal and variable retention. Genetic patterns can be patchy and inconsistent across cohorts and may not be captured in a single sampling year or when samples are combined [6,7]. By focusing on juveniles, which represent discrete recruitment events, temporal sampling allows for more precise inference of cohort-specific structure and connectivity in marine species characterized by high dispersal potential.

Temporal population genomic studies can provide insight into recruitment and spawning stock dynamics in marine fishes. These dynamics include temporal fluctuations in reproductive success among spawning groups, including sweepstakes reproductive events, variable larval survival, and shifting contributions from different spawning locations or cohorts [6,8]. From a management perspective, this information is essential for understanding how the breeding population contributes to recruitment, evaluating the stability of spawning stock composition, and assessing whether local populations function as discrete units or are replenished by variable sources over time. Consequently, incorporating temporal genomic data strengthens stock assessment and informs management strategies by linking genetic patterns directly to recruitment variability and spawning stock dynamics.

Southern flounder (*Paralichthys lethostigma*) is a widely distributed coastal flatfish occurring throughout the U.S. Southeast Atlantic and Gulf of Mexico [9]. The species exhibits life history traits common to marine fishes, including offshore spawning and a pelagic larval stage, which promote high dispersal and connectivity across its range [10]. Previous genetic studies using allozymes/microsatellites and mtDNA/AFLPs supported strong Atlantic–Gulf differentiation and weak or inconsistent structure within the U.S. Atlantic, with inferences largely drawn from broad spatial sampling of only a few sites at one point of time [11,12]. A genetic analysis using mitochondrial DNA and AFLPs further identified weak structure and evidence of basin-wide mixing within the U.S. Atlantic, without explicit evaluation of temporal stability [13]. However, a recent study applying next-generation sequencing approaches found some evidence of estuarine-scale structure in southern flounder in the Gulf and Atlantic using samples pooled across years and found that genetic heterogeneity may be driven by environmental variation rather than geographic distance [14]. However, whether fine-scale genetic structure is present and consistently detectable across estuaries through time remains unresolved in both the Gulf and Atlantic populations. Understanding fine-scale and potentially transient genetic structure in southern flounder requires grounding genetic patterns within the context of the species’ life history, particularly the processes that govern larval dispersal and estuarine recruitment.

Southern flounder exhibit a life history typical of estuarine-dependent flatfishes, with discrete life stages that structure both dispersal potential and population connectivity. Adults migrate from estuarine habitats to offshore waters in the late fall and early winter [15,16], where batch spawning occurs from approximately November through January in the Gulf of Mexico [17] and October through December in the Southeast U.S [18]. Following hatching, larvae undergo a pelagic phase of approximately 30–60 days on the outer continental shelf before transitioning towards inshore estuaries [18]. Juvenile flounder congregate near inlets and barrier island surf zones, settling in estuarine habitats in late winter and early spring following metamorphosis from the larval stage [19–21]. Juveniles and sub-adults reside in estuaries for approximately one year until they reach sexual maturity, after which individuals migrate offshore to spawn. While most adults return to estuaries after spawning, some remain in open coastal waters year-round [9]. This life history creates conditions under which fine-scale, temporally variable genetic structure may arise among estuarine cohorts (i.e., juveniles that settle together at a location within a settlement year).

To address this, we assess fine-scale and temporal patterns of population genetic structure in juvenile southern flounder in North Carolina and Texas estuaries using high-resolution genomic data obtained through ddRAD sequencing, providing insight into genetic patterns associated with recent recruitment. We chose to focus primarily on North Carolina, a region with relatively few fine-scale population genetic studies and a spatially complex intracoastal structure. Specifically, we examine whether genetic differentiation is detectable within and among estuaries and whether these patterns are consistent across multiple sampling years. Given the species’ high dispersal potential, we expect that fine-scale genetic structure would be weak and temporally variable. By evaluating fine-scale genetic structure across space and time, this study informs key assumptions about connectivity in highly dispersive marine species within heterogeneous estuarine systems and supports the delineation of biologically relevant management units.

## Materials and methods

### Sample locations

**Fig 1:**
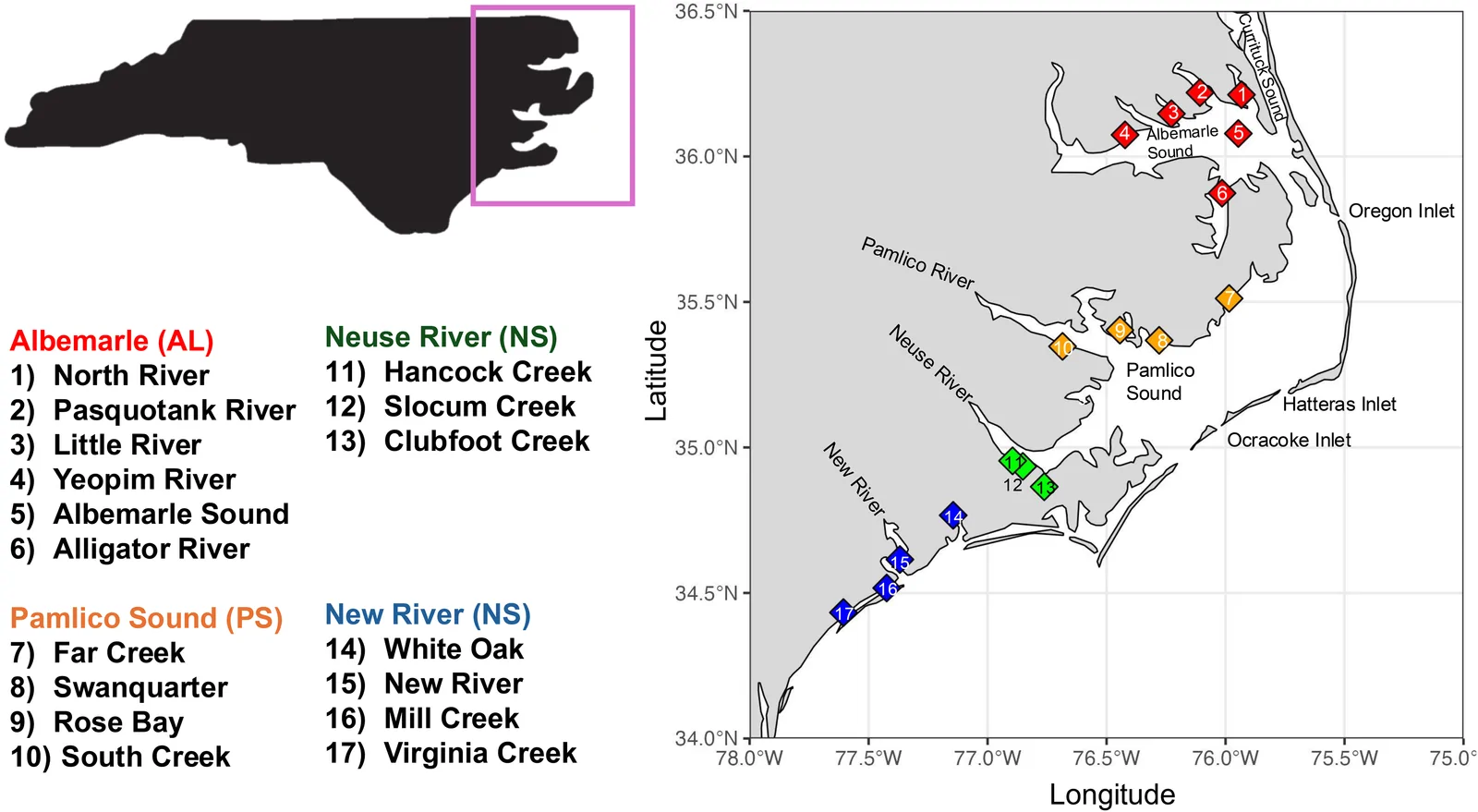
Map of sampling locations for southern flounder (*Paralichthys lethostigma*) in North Carolina estuaries. The inset highlights the study region within North Carolina. Sampling sites are grouped into four regions: Albemarle (AL; sites 1–6), Pamlico Sound (PS; sites 7–10), Neuse River (NS; sites 11–13), and New River (NR; sites 14–17). Numbered markers correspond to individual sampling locations listed in the legend and are color-coded by region.

**Fig 2:**
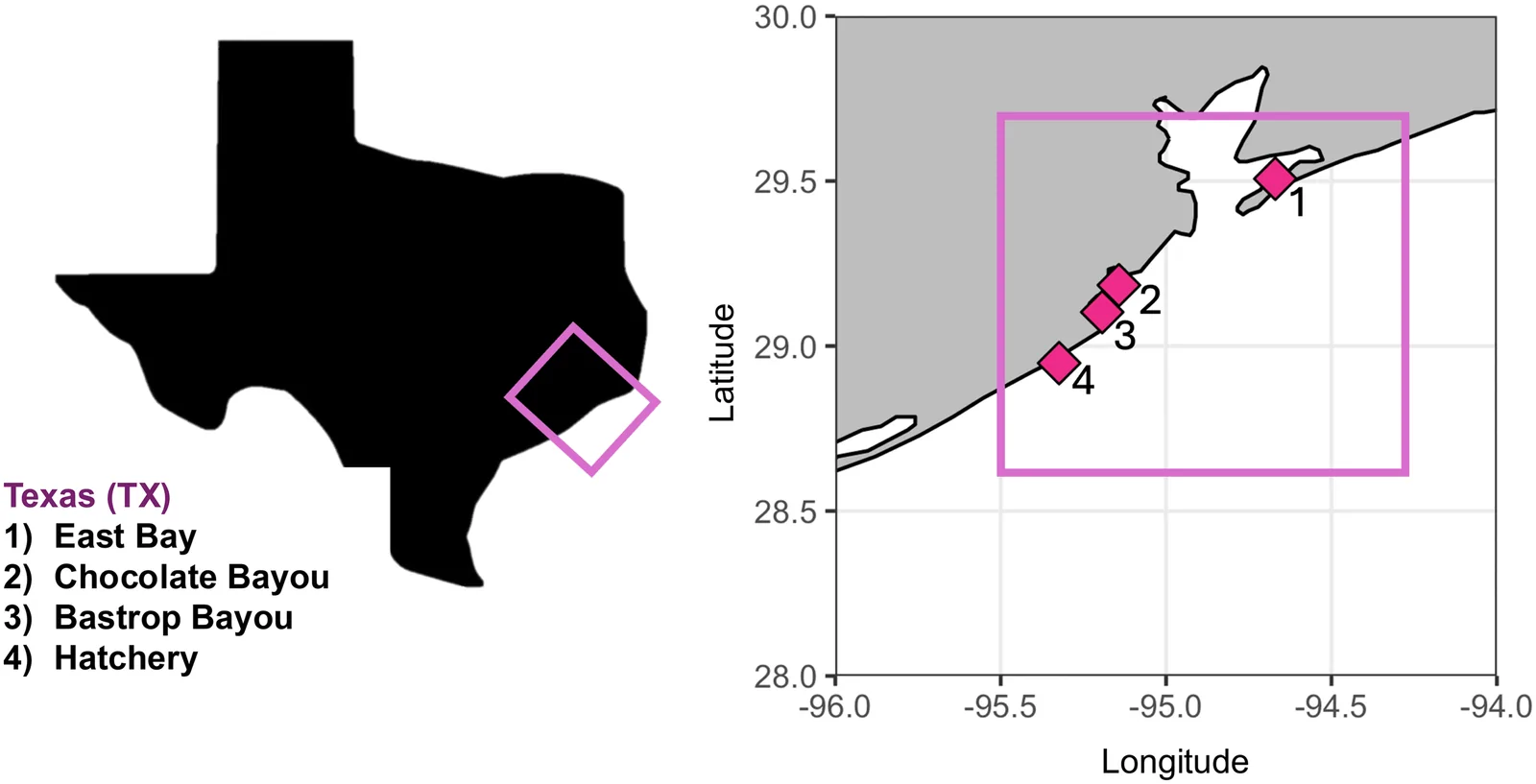
Map of sampling locations for southern flounder (*Paralichthys lethostigma*) in Texas. The inset highlights the study region along the upper Texas coast near Galveston Bay. Sampling sites include (1) East Bay, (2) Chocolate Bayou, (3) Bastrop Bayou, and (4) a hatchery population. Numbered markers correspond to the sampling locations shown on the map.

Samples for this study were collected from two Ocean Basins: The Southeast U.S. Atlantic (North Carolina; Fig 1) and the Gulf of Mexico (Texas; Fig 2). We sampled juvenile fish from estuarine environments where larval flounder settle and remain for approximately 1–2 years before moving offshore for spawning [9]. Estuarine sampling locations within North Carolina were grouped into three regions — Pamlico Sound, Neuse River, and New River — based on geographic barriers and environmental differences.

Each of the four regions in North Carolina are affected by different environmental and spatial factors (Fig 1). Pamlico Sound is an enclosed estuary system with the barrier islands to the East. Pamlico Sound has the lowest and most stable salinity and temperature of the regions due to limited tidal exchange and large freshwater inputs [22]. While the Neuse River, our intermediate region, is connected to the ocean via Pamlico Sound, it has a higher rate of flushing than Pamlico and is more variable in temperature and salinity [22,23]. Our southernmost region, the New River region (herein, New River), experiences higher average salinity and temperature, with greater fluctuations driven by tidal input and less freshwater flow [22–24]. In 2023, our expanded sampling included Albemarle Sound, a northern region with environmental conditions more closely aligned with Pamlico Sound; however, Albemarle Sound differs from Pamlico Sound with lower average temperatures, higher variation in freshwater inflow, and a single narrow inlet (the Oregon Inlet) connecting the Sound to the Atlantic Ocean [25]. As sampling occurred in different locations from year-to-year, the sampling locations within each region vary. Sampling in 2022 was limited to two locations in the Neuse River region; these samples were therefore incorporated into temporal analyses rather than treated as an independent sampling year.

### Sample collection

We obtained juvenile southern flounder from North Carolina Division of Marine Fisheries (NCDMF) biologists during the juvenile monitoring Program 120 (P120) estuarine trawl survey in 2014−2016, 2022, and 2023 (S1 Table). The survey samples shallow, upper estuary locations using an otter trawl with a 3.2 m headrope, 6.4 mm bar mesh wings and body, and a 3.2 mm bar mesh cod end. The trawl is towed for 1 min at 1.1 m/s at each station. Sampling locations within four geographic regions are shown in Fig 1. In 2016, we supplemented NCDMF sample collection with our own sampling using an otter trawl or 2-meter-wide beam trawl depending on habitat [23]. Fish were frozen upon capture and stored at −20 C prior to DNA extraction. Total lengths of sampled individuals ranged from approximately 35–75 mm, consistent with young-of-year juveniles representing a single newly-recruited year-class. Therefore, we excluded individuals that were from previous year-classes. The research was approved and performed in accordance with the relevant guidelines and regulations by the Institutional Animal Care and Use Committees at North Carolina State University (#13-139-0, 17-003-0). Scientific Collection Permits for capture of wild flounder were obtained from the North Carolina Division of Marine Fisheries, Morehead City, NC.

To assess broad genetic structure between ocean basins for new year-classes, we included juvenile samples collected in Texas in our analysis. We obtained Texas samples from Texas Parks and Wildlife in 2014, 2015 and 2016 from three locations: East Bay, Bastrop Bayou and Chocolate Bayou (Fig 2). Fish were sampled using 1- and 2-meter-wide beam trawls pulled for 1–2 minutes at 1–2 knots. In areas where boat sampling was restricted, fish were sampled using trawls pulled by-hand for 20 meters. We also included samples from the Sea Center Texas Marine Fish Hatchery (Lake Jackson, TX) in our 2016 data set to assess the genetic similarity between hatchery and wild-caught Texas individuals, with implications for stock enhancement programs. Information on the geographic origin of hatchery broodstock was not available for this study. A total of 333 juveniles were sampled across all regions and years, ranging from seven to 61 individuals per sampling group (Table 2).

### Extraction, sequencing and genomic library building

We extracted genomic DNA from fin clips using Qiagen DNA Blood and Tissue kits, following the manufacturer’s instructions (Qiagen, Inc., Valencia, CA) and quantified total genomic DNA with a Qubit fluorometer 4.0 (Invitrogen, Carlsbad, CA). We prepared ddRAD sequencing libraries with enzyme pair *SphI* and *MluCI* following the protocol detailed by Burford Reiskind et al. (2016), and digested genomic DNA with both enzymes in CutSmart buffer at 37°C for 3 hours. We then ligated adapters using T4 ligase, and incubated ligation reactions at 22°C for 2 hours followed by heat inactivation at 65°C for 20 minutes. Each individual fish had specific barcodes, incorporated during adapter ligation, allowing each individual to be identified and demultiplexed following sequencing. We constructed libraries containing 48 individuals and purified each library using the Qiaquick PCR cleanup kit (Qiagen, Inc., Valencia, CA). After cleanup, we size-selected each library for fragments between 360–475 bp using a BluePippin system (Sage Science, Beverly, MA). Following size selection, libraries were amplified and sequenced as single-end 90 bp fragments on the Illumina NovaSeq at the North Carolina State University Genomic Sequencing Laboratory (Raleigh, NC, USA). After sequencing, each individual was de-multiplexed based on individual barcodes and data was retained for each individual fish.

### Bioinformatic pipeline

We used the program FastQC (Babraham Bioinformatics; http://www.bioinformatics.babraham.ac.uk/projects/fastqc/) to check the quality of reads, using a high phred score criterion (phred >33). We then processed barcodes as outlined in Burford Reiskind et al. [26] using the *process_radtags* pipeline to filter and de-multiplex variable length barcodes in STACKS v. 1.24 [27]. This results in a folder for each individual juvenile and their associated reads. We trimmed reads to identical lengths (90 bp) and used the *de novo* pipeline (*denovo.pl*) in STACKS to detect single nucleotide polymorphisms (SNPs) with the following parameters: *m* = 3 (minimum stack depth), *M* = 2 (mismatches allowed between loci within an individual), and *n* = 2 (mismatches allowed between loci combined in a catalog; [27]). From the STACKS *de novo* pipeline, we identified 3,643,301 loci across all North Carolina and Texas samples with an average per-sample coverage of 22.2x. A reference genome was not available for this study, but *de novo* approaches reliably capture patterns of genetic differentiation in non-model organisms [27].

Because a congener summer flounder (*P. dentatus*) overlaps in its range with southern flounder, and the juveniles are morphologically similar, we confirmed that all sampled individuals were *P. lethostigma* using Treemix v. 1.13 [28] to produce maximum-joining trees for each sampling year. To confirm branching patterns, we conducted a bootstrap analysis using 1000 replicates. We identified four individuals in 2023 that belonged to *P. dentatus.* These individuals were removed from the dataset.

### Dataset filtering and transformation

We ran STACKS *populations* pipeline retaining loci present in at least one (-p 1) or two (-p 2) populations and requiring a minimum within-group genotyping rate of 60% (-r 0.6). We retained one SNP per rad locus (--write-random-snp) and excluded loci with high heterozygosity (--max-observed-het 0.8).

We then filtered in PLINK (PLINK v.1.19; http://pngu.mgh.harvard.edu/purcell/plink/) [29] for minor allele frequency (maf 0.01) and locus missingness (geno 0.5). Because different analyses require different assumptions of locus sharing and data completeness, we generated multiple SNP datasets optimized for specific objectives. Within each dataset, individuals with excessive missing data were removed using the –mind filter, with thresholds ranging from 0.3–0.5 depending on the sequencing quality of each sampling year; higher thresholds were applied to years with lower overall sequencing quality.

After filtering in PLINK, we transformed the datasets into genetix files for further analysis using PGDSpider (v 2.1.1.0) [30]. We further filtered SNP datasets for deviations from Hardy Weinberg Equilibrium via the “out-all” method (removes loci that deviate from HWE in all populations) using the R package *DartR* [31].

### Texas vs. North Carolina

To identify broad-scale genetic differentiation between Texas (TX) and North Carolina (NC), we analyzed all individuals from both states grouped by sampling year (e.g., NC 2014, TX 2015). We then partitioned by year (i.e., TX vs. NC 2014, TX vs. NC 2015, TX vs. NC 2016).

We calculated pairwise measures of genetic differentiation (*F*_*ST*_) using the R package *hierfstat* (v. 05–11; [32]) for each sampling year (2014–2016) using the command “pairwise.WCfst” [33]. We then used “boot.ppfst” to calculate *F*_ST_ confidence intervals (CI = 0.025, 0.975) using 1000 bootstraps. The “boot.ppfst” function repeatedly resamples loci to generate empirical distributions of pairwise *F*_ST_ estimates. We considered pairwise *F*_*ST*_ values statistically significant when the 95% confidence interval did not overlap zero, indicating differentiation exceeding that expected by chance given sampling variation.

### Texas

The dataset that included all Texas locations and years consisted of 38,013 loci with an overall genotyping rate of 0.90. We then analyzed each sample year separately (2014, 2015, 2016).

For each year, we calculated genetic summary characteristics, including observed heterozygosity (*H*_*o*_), expected heterozygosity (*H*_*E*_) and the inbreeding coefficient (*F*_*IS*_), using the *basic.stats* function in *hierfstat* [32].

To identify genetic clusters and estimate cluster membership probabilities within Texas, we performed principal components analysis (PCA) for each sampling year in R using the packages *adegenet* (v. 1.3–1; [34]) and *Ade4* (v. 1.7–23; [35]). We used principal components analysis (PCA) to determine clustering as it does not require *a priori* group assignment. PCA summarizes genetic variation among individuals, with individuals that are more genetically similar plotting closer together. Distinct clusters or dispersion along principal component axes indicate genetic differentiation among groups*.* We retained the first two principal components of each PCA because they explained the greatest proportion of genetic variance and no additional structure was apparent in subsequent components. We then produced scatter plots using *ggplot2* in R [36].

For the one location in Texas, Bastrop Bayou, that had yearly samples (2014, 2015, and 2016), we calculated pairwise *F*_*ST*_ values in *hierfstat* and conducted a PCA in *adegenet* to assess temporal differentiation within this location.

### North Carolina

To investigate population structure among juvenile flounder within NC, individuals were grouped by location and year. We ran the *populations* pipeline in STACKS with a stricter locus sharing criterion (-p 2) to remove loci present in only a single population and reduce the influence of rare or location-specific variants. After filtering, the NC dataset consisted of 25,085 loci. To produce genetic summary statistics, we grouped the dataset by each region (Albemarle, Pamlico Sound, Neuse River, New River) and year, and calculated statistics using *basic.stats* in *hierfstat* [32].

Then, to determine population genetic structure among specific locations in NC, we grouped the dataset by specific sampling location and year and used this dataset to calculate pairwise *F*_*ST*_ values. Although some sampling locations within NC contained a small number of individuals (n = 4–5), this limitation is mitigated by the large number of SNPs analyzed (25,085). Previous studies have shown that increasing the number of loci improves the accuracy of population genetic inference and can compensate for a small number of individuals [37–39]. To confirm patterns of genetic structure, we calculated pairwise *F*_*ST*_ among combined regions and years (Albemarle, Pamlico Sound, Neuse River, New River), which increased sample sizes by pooling individuals from multiple locations within each region (S4 Table). We also conducted multivariate and clustering analyses including principal components analysis (PCA) and ADMIXTURE (v. 1.3; [40]) for specific NC sampling locations and years. To determine the optimal value for K in the ADMIXTURE model, we generated standard error scores using cross-validation for K = 1-n (where n is the total number of *a priori* sampling locations in the dataset) and selected the K value with the lowest cross-validation error. We generated plots in R using *ggplot2* [36] from ADMIXTURE Q matrices with individuals ordered North to South by location.

To assess temporal genetic structure within regions, we analyzed locations sampled in at least three years. These locations included: one location in the Pamlico Sound Region – Swanquarter (2014, 2015, 2016); three locations in the Neuse River Region – Hancock Creek (2014, 2016, 2022, 2023), Slocum Creek (2014, 2016, 2023), and Clubfoot Creek (2014, 2016, 2022, 2023); and two locations in the New River Region – Mill Creek (2014, 2015, 2016) and Virginia Creek (2014, 2016, 2023). We calculated pairwise *F*_*ST*_ for each temporal dataset in *hierfstat* and produced a PCA in *adegenet*. All custom scripts used for data processing and analysis are publicly available at https://doi.org/10.5281/zenodo.21168058.

## Results

We completed genetic analyses on a total of 266 juvenile southern flounder collected across five sampling years (2014–2016, 2022, and 2023) from 17 estuarine locations in four regions of North Carolina. An additional 67 individuals were analyzed from three coastal locations and one hatchery population in Texas, sampled between 2014 and 2016.

### Broad geographic-scale differentiation - North Carolina and Texas

We found consistent, and significant genetic differentiation between juvenile southern flounder populations from Texas and North Carolina in all three sampling years (*F*_*ST*_ values = 0.0467 (2014), 0.0542 (2015) and 0.0620 (2016); S2 Table). All values were significant based on confidence intervals produced with 1000 bootstraps.

### Texas

#### Genetic characteristics.

Genetic diversity in Texas was similar across the limited sampling years (2014–2016). We found expected heterozygosity (*H*_*E*_) ranged from 0.1224 to 0.1362 and all years showed slightly positive inbreeding coefficients (*F*_*IS*_ = 0.0949 to 0.1060; Table 1). A slight deficit of heterozygotes relative to Hardy-Weinberg expectations was observed across all sampling groups (mean *H*_*O*_ = 0.117, mean *H*_*E*_ = 0.130).

**Table 1:** Genetic statistics for Texas samples by year.

	N	H_O_	H_E_	F_IS_
**Texas 2014**	34	0.1218	0.1362	0.1060
**Texas 2015**	24	0.1108	0.1224	0.0952
**Texas 2016**	9	0.1185	0.1309	0.0949

N represents the number of individuals sampled, Ho is observed heterozygosity, He is expected heterozygosity, and Fis is the inbreeding coefficient calculated from genome-wide SNP data for 2014–2016.

#### Spatial structure in Texas.

**Fig 3:**
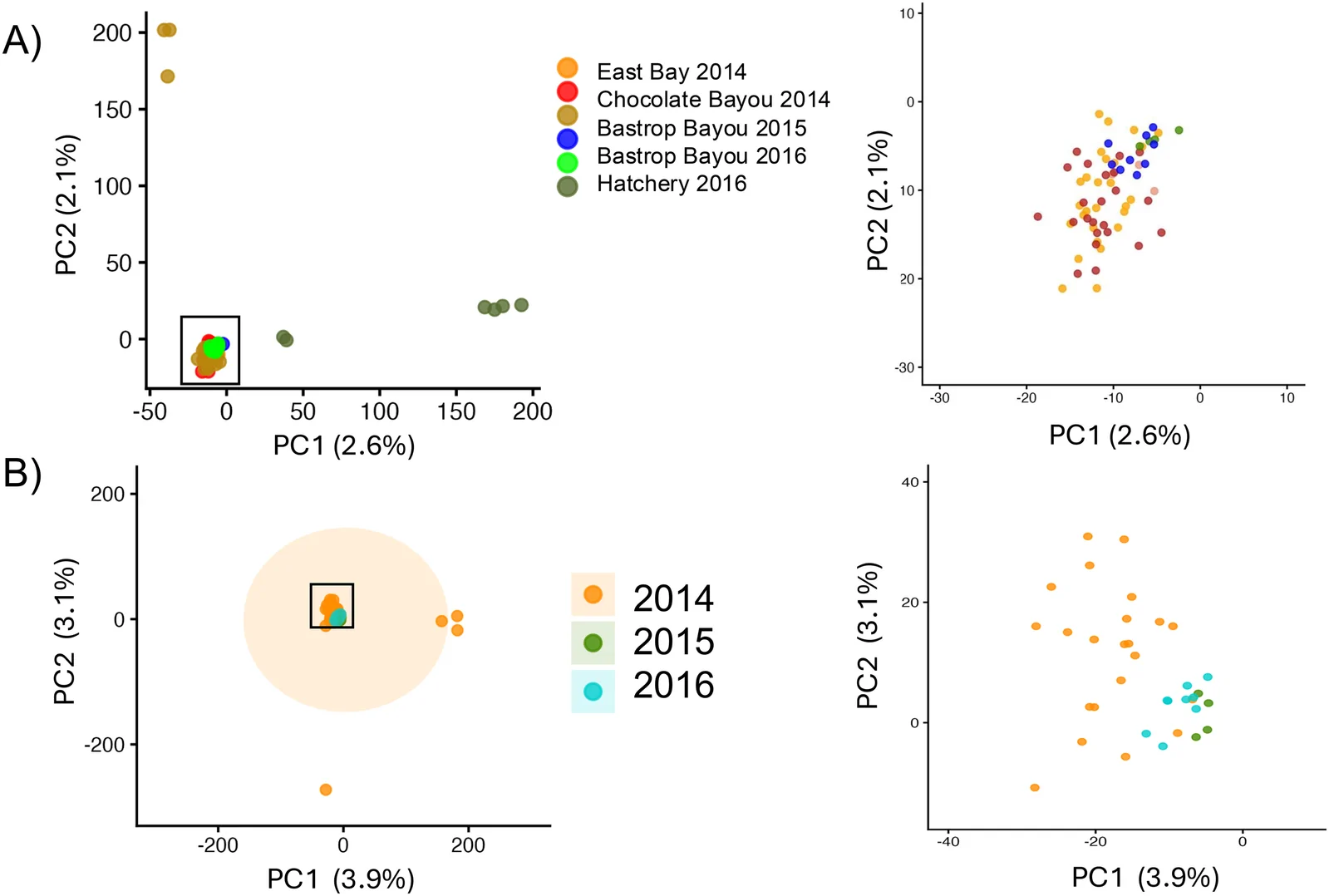
Principal component analysis (PCA) of southern flounder (*Paralichthys lethostigma*) from Texas. (A) PCA including all sampled locations and years: East Bay (2014), Chocolate Bayou (2014), Bastrop Bayou (2014–2016), and hatchery (2016). (B) PCA including only Bastrop Bayou samples from 2014–2016. Left panels show the full PCA, and right panels show a magnified view of the central cluster (boxed). Points represent individuals and are colored by location (A) or year (B). Axes indicate the percent variance explained by each principal component. Shaded ellipses indicate 95% confidence intervals around the centroid for each sampling year.

The results of the PCA that analyzed genetic variation among individuals showed limited genetic differentiation among most Texas sampling locations and years. The first two principal components of the Texas PCA explained 2.6% and 2.1% of the total genetic variation (Fig 3A). Individuals from East Bay (2014), Chocolate Bayou (2014), and Bastrop Bayou (2014–2016) largely overlapped near the center of the plot, indicating little genetic structuring among these sites or across Bastrop Bayou sampling years. In contrast, individuals from the 2016 hatchery population formed a distinct cluster separated from the wild samples along PC1. The hatchery individuals had a slightly lower genotyping rate (0.83) than wild samples, which may have contributed to their distinct positioning in the PCA in addition to genuine genetic differentiation.

#### Temporal structure in Bastrop Bayou.

The temporal PCA of Bastrop Bayou, TX, showed that 2015 and 2016 clustered tightly together, while 2014 showed a high degree of individual variation along both the PC1 and PC2 axes (3.9% and 3.1%; Fig 3B), with four of nine individuals falling outside the central cluster. Although 2015 and 2016 individuals clustered tightly in the PCA, we found significant differentiation between 2015 and 2016 based on pairwise *F*_*ST*_ analysis (S8G Table). This likely reflects the greater sensitivity of genome-wide allele frequency comparisons relative to visual PCA clustering for detecting subtle genetic variation. *F*_*ST*_ values ranged from 0.0002–0.0035, and we found significant differences between 2015 and 2016 (S8G Table).

### North Carolina

#### Regional genetic characteristics.

Genetic diversity was similar across regions and years (Table 2). Expected heterozygosity (*H*_*E*_) ranged from 0.1413 to 0.1614, with the highest value observed in Neuse River (2016). Across all populations, *H*_*E*_ was slightly higher than *H*_*O*_, resulting in consistently positive inbreeding coefficients (*F*_*IS*_ = 0.070 to 0.105).

**Table 2:** Genetic statistics for North Carolina samples by region and year.

	N	H_O_	H_E_	F_IS_
**Albemarle 2023**	61	0.1407	0.1565	0.1013
**Pamlico 2014**	21	0.1480	0.1603	0.0763
**Pamlico 2015**	22	0.1330	0.1454	0.0856
**Pamlico 2016**	9	0.1485	0.1604	0.0745
**Neuse 2014**	20	0.1474	0.1585	0.0701
**Neuse 2015**	26	0.1294	0.1413	0.0842
**Neuse 2016**	29	0.1489	0.1614	0.0778
**Neuse 2023**	12	0.1462	0.1581	0.0757
**New River 2014**	18	0.1488	0.1605	0.0728
**New River 2015**	7	0.1519	0.1519	0.0799
**New River 2016**	26	0.1479	0.1600	0.0752
**New River 2023**	15	0.1544	0.1544	0.1053

N represents the number of individuals sampled, H_O_ is observed heterozygosity, H_E_ is expected heterozygosity, and F_IS_ is the inbreeding coefficient calculated from genome-wide SNP data for 2014–2016 and 2023.

#### Overall spatial structure in North Carolina by year.

Within North Carolina, we found significant genetic differentiation that varied among sampling locations and years for almost all pairwise comparisons among broad regions (S7 Table). Full pairwise *F*_ST_ matrices are found in supplementary S3-S6 Tables. Although the observed pairwise *F*_ST_ values were small in magnitude, these values were expected given the marker type in this study and supporting consistent but spatially variable genetic structure in this region.

**Fig 4:**
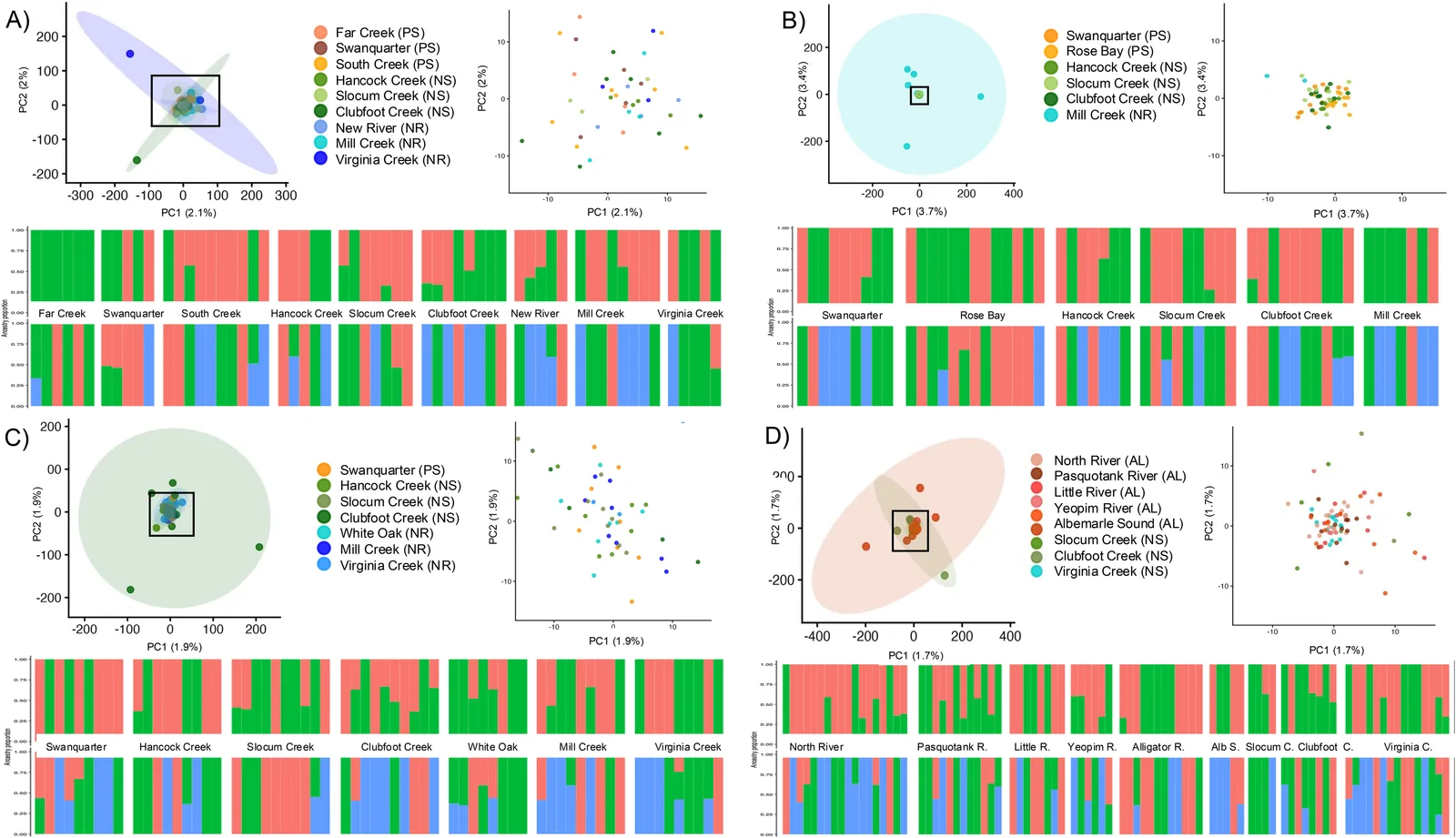
Population structure of southern flounder (*Paralichthys lethostigma*) sampled from North Carolina estuaries across four years. (A) 2014, (B) 2015, (C) 2016, and (D) 2023. For each year, the upper panels show a principal component analysis (PCA) of individuals by sampling location, with the full PCA on the left and a magnified view of the central cluster (boxed) on the right. Axes indicate the percent variance explained by each principal component. The lower panels show admixture results for K = 2(top) and K = 3(bottom), with each vertical bar representing an individual and colors indicating inferred ancestry proportions. Note that the magnified panels show a subset of individuals for clarity; PC axes reflect variance explained in the full dataset.

To better understand these patterns, we examined the genetic structure within each year. In 2014, we did not find significant pairwise *F*_ST_ values (S3 Table), and the PCA revealed minimal genetic clustering among sampling locations (Fig 4A). The first two components of the PCA explained 2.1% and 2.0% of the total genetic variance, respectively. Individuals from most populations showed substantial overlap in multivariate space, indicating limited genetic differentiation. In the New River Region, one individual from Virginia Creek showed notable divergence along PC2 relative to other populations.

In 2015, we found significant pairwise *F*_ST_ values at five of the 15 comparisons (S4 Table), which was supported by the results of the PCA. Of the five significant comparisons in the pairwise *F*_ST_ analysis, four included the Mill Creek site in the southernmost New River Region. We also found significant pairwise *F*_*ST*_ between two sites in Pamlico Sound: Rose Bay and Swanquarter. The results of the PCA in 2015 explained 3.7% and 3.4% of variation, respectively (Fig 4B). We found notable overlap of all sampling sites except samples from Mill Creek, which were highly dispersed across the ordination space and included several individuals strongly diverged from all other populations in the PCA.

In 2016, we did not find significant pairwise *F*_*ST*_ values (S5 Table), and the results of the PCA supported a lack of spatial population genetic structure as most sampling sites clustered tightly (Fig 4C). However, the Clubfoot Creek location did include a few individuals that dispersed along PC1 and PC2. Both axes explained 1.9% of the total genetic variance.

In 2023, we included samples from Albemarle Sound Region, which was the northernmost region sampled. With the addition of this northernmost region, we found greater genetic differentiation among sites. We found significant pairwise *F*_ST_ values at 13 out of 36 comparisons (S6 Table). Of these 13 comparisons, six were between Albemarle and Neuse locations and seven were among sample locations within Albemarle. The results of the PCA showed both axes explained 1.7% of the total variance (Fig 4D). While most individuals from all sampling locations clustered tightly, samples from Albemarle Sound and Clubfoot Creek (Neuse) showed some individual divergence along the PC1 and PC2 axes.

The results of the ADMIXTURE analysis were similar among all years (Fig 4A-D). Across all years, cross-validation (CV) error was lowest at K = 1 and increased at higher K values, indicating that a single undifferentiated population best explains the data and is consistent with panmixia. Although cross-validation supported K = 1 in all years, admixture plots K = 2 and K = 3 are shown for visual reference to illustrate the distribution of individual-level ancestry variation, though these values are not supported by cross-validation and should be interpreted cautiously. The individual-level variation at higher K values should be interpreted in the context of the cross-validation results, which indicate that this variation does not reflect discrete population structure among sampling locations.

#### Temporal variation of specific NC sampling sites.

When we analyzed year to year variation within a site, we found significant genetic differences across years within sites in our pairwise *F*_*ST*_ analysis that was consistent with the results of our PCAs.

**Fig 5:**
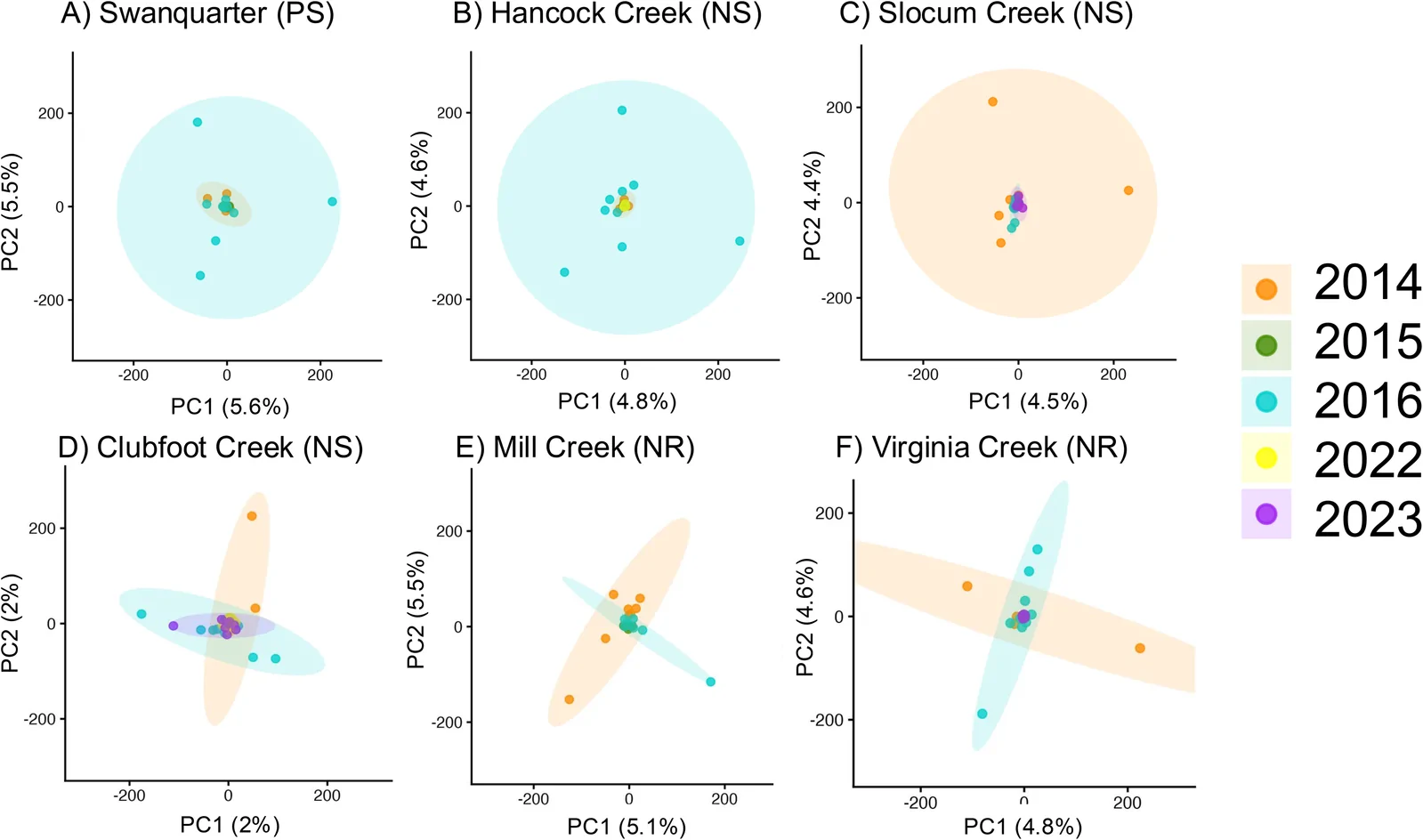
Principal component analyses (PCA) of southern flounder (*Paralichthys lethostigma*) used to examine temporal population structure within North Carolina estuaries. Sampling locations are arranged geographically from north to south (top left to bottom right): (A) Swanquarter (Pamlico Sound; PS), (B) Hancock Creek (Neuse River; NS), (C) Slocum Creek (Neuse River; NS), (D) Clubfoot Creek (Neuse River; NS), (E) Mill Creek (New River; NR), and (F) Virginia Creek (New River; NR). Points represent individuals colored by sampling year (2014, 2015, 2016, 2022, and 2023), with ellipses indicating dispersion within years. Axes show the percent variance explained by the first two principal components.

*Pamlico Region*. For Swanquarter, we found significant genetic differentiation between years in our pairwise *F*_*ST*_ analysis at two of the three comparisons: 2015 compared to either 2014 (*F*_*ST*_ = 0.0056) or 2016 (*F*_*ST*_ = 0.0027; S8A Table). For the PCA, we found PC1 and PC2 explained 5.5% and 5.6% of the variation. Samples from 2014 and 2015 formed a tight cluster, while 2016 samples showed slightly greater dispersion along both PC1 and PC2 (Fig 5A).

*Neuse River Region.* In this region we had three sites that had multiple years sampled. We found significant genetic differentiation among years at Hancock Creek at four of six pairwise comparisons (*F*_*ST*_  = 0.0014–0.0040; S8B Table). The PCA (PC1: 4.8%, PC2: 4.6%) showed overlap between 2014, 2015 and 2022, while 2016 individuals clustered separately showing high divergence. We also found significant genetic differentiation among years in Clubfoot Creek at eight of ten pairwise comparisons (*F*_*ST*_ = 0.0016–0.0036; S8C Table). We found greater divergence among individuals at Clubfoot Creek compared to Hancock Creek in the PCA analysis (Fig 5). The Clubfoot Creek PCA (PC1: 2%, PC2: 2%) showed that, while most individuals cluster tightly, 2014, 2015, and 2016 all contain highly differentiated individuals, with 2016 showing the highest degree of divergence. At the third location, Slocum Creek, we found significant genetic differentiation among years at five of the six pairwise *F*_*ST*_ comparisons (*F*_*ST*_ = 0.0027–0.0035; S8C Table), and the PCA (PC1: 4.5%, PC2: 4.4%) showed 2015, 2016 and 2023 clustered tightly with some separation between 2016 and 2023. In addition, 2014 contained highly divergent individuals on both axes.

*New River Region*. In this southernmost region, we found greater genetic structure among years at the two sites we had yearly sampling than the other two regions. At Mill Creek we found significant genetic differentiation among years in two of three pairwise *F*_*ST*_ comparisons (0.0031–0.0039; S8E Table). The PCA showed that 2023 clustered together tightly, while 2014 and 2016 contained highly divergent individuals that clustered on separate axes (Fig 5E). In Virginia Creek, no pairwise comparisons were significant (S8F Table). The PCA showed a similar pattern to Mill Creek, with 2015 clustered tightly, while 2014 and 2016 showed higher divergence on separate axes.

## Discussion

Our study evaluated spatial and temporal genetic structure of juvenile southern flounder from two different basins in its range, specifically in Texas and North Carolina, with a particular emphasis on the poorly studied North Carolina region in the Atlantic where there has been a substantial decline in flounder populations [9]. Previous studies on the southern flounder have either pooled samples across years or lacked fine-scale spatial sampling at scales likely corresponding to the contributions from individual spawning stocks. By incorporating both broad and fine spatial scales and sampling across multiple years, we aimed to capture recruitment patterns associated with discrete yearly cohorts of settled juveniles in estuarine locations rather than signals averaged across multiple cohorts. This approach enabled us to resolve spatial and temporal variation in spawning stock contributions among estuaries and years.

At a broad scale, our analyses revealed clear genetic differentiation between Texas and North Carolina, consistent with previous studies documenting large basin genetic structure between Gulf of Mexico and Atlantic populations [12–14]. Within Texas and North Carolina, we detected fine-scale genetic structure that varied in strength across sampling years, suggesting temporal shifts in the genetic composition of recruiting juveniles from the adult spawning stock. In North Carolina, we observed year-to-year variation in genetic composition among individuals within sampling sites, indicating that local nurseries may receive recruits from multiple spawning cohorts. Together, our findings highlight the importance of including both spatial and temporal sampling when evaluating population structure to more effectively resolve spawning stock contributions to estuarine recruitment. We use the term ‘spawning stock’ broadly to refer to the collective group of adults contributing to a given recruitment cohort, rather than implying discrete spatial aggregations of spawning individuals. The spatial organization of southern flounder spawning behavior remains poorly understood. While volitional spawning has been observed in aquaculture settings [41,42], spawning aggregations have not been documented in the wild, and it remains unclear whether adults migrate to discrete spawning locations or spawn more broadly across offshore habitats. Our Gulf sampling was limited to sites within Texas, therefore, these temporal differences support the existence of fine-scale, dynamic population structure within the Gulf of Mexico, consistent with the genetic heterogeneity previously reported by O’Leary et al. [14]. The oceanographic complexity of the Gulf of Mexico, including wind-driven circulation, freshwater influx, and mesoscale eddies, provides a plausible mechanism for this temporal variability [43].

### Broad-scale differentiation between North Carolina and Texas

The significant genetic differentiation between southern flounder from different basins, the Atlantic (North Carolina) and Gulf of Mexico (Texas), supports earlier studies using otolith chemistry, microsatellites, AFLPs, and SNPs, all of which suggest the Gulf and Atlantic represent distinct stocks [12–14]. Anderson et al. [12] proposed that this divergence reflects independent post-Last Glacial Maximum (~20,000 years ago) expansion of southern flounder populations in the Gulf of Mexico and Atlantic, resulting in long-term isolation between basins. This Atlantic-Gulf split is a common pattern among coastal marine species, including red drum, an estuarine-dependent species with comparable life history traits [44]. Currently, Atlantic and Gulf flounder are managed as separate stocks, and our findings provide additional support for maintaining this management framework.

### Regional differentiation in Texas

Within Texas, we did not find significant genetic divergence among sites within a year; however, samples from Bastrop Bayou exhibited significant interannual genetic differentiation suggesting that larval recruitment or spawning stock composition at this location varies among years.

Our analysis revealed that individuals from the hatchery were genetically distinct from wild-caught juveniles, suggesting that the hatchery broodstock may not fully represent the local spawning stock. This is likely due to founder effects, reduced genetic diversity associated with a limited founding population, and/or non-local sourcing. While information on broodstock origin was not available for this study, the genetic diversity (*H*_*E*_) was not significantly reduced relative to wild populations and likely reflects a distinct lineage rather than a recent bottleneck. While the introduction of genetically distinct individuals could, in some cases, increase genetic diversity in wild populations, it could also lead to outbreeding depression. Such outcomes depend on the composition of the hatchery stock and the degree of selection for hatchery conditions. Hatchery lines affected by founder effects or selection may be maladapted to natural environments, and their introduction can reduce the overall fitness of the population they are introduced to (outbreeding depression). Similar challenges have been documented in other fish species such as salmonids [6,45], where hatchery releases led to reduced fitness or altered population structure in wild populations. These results highlight the importance of carefully evaluating broodstock sourcing and management in stock enhancement programs to ensure that efforts support the genetic integrity and adaptive potential of wild southern flounder populations.

Overall, our results suggest a pattern of high connectivity within Texas accompanied by localized and temporally variable genetic structure, likely driven by recruitment processes rather than persistent barriers to gene flow. While most studies have found limited genetic differentiation in the Gulf of Mexico [12], some evidence supports the presence of finer-scale structure [13]. Both studies included a mixture of life stages (i.e., juveniles and adults or multiple age classes) which may integrate signals across cohorts and years and therefore reflect a time-averaged view of connectivity. In contrast, our focus on juveniles likely enhances the detection of cohort-specific genetic structure. Although our sampling was limited to Texas and did not reveal consistent large-scale structure, dynamic patterns may occur elsewhere in the Gulf. Expanded spatial and temporal sampling will be necessary to more fully resolve southern flounder population dynamics across this region.

### Regional differentiation in North Carolina

Within North Carolina, we found significant fine-scale genetic structure among estuarine sites that varied among years. Some individual sampling locations showed greater genetic divergence across years than other sampling locations within the same year, indicating temporal variability in population composition. This is supported by significant pairwise *F*_*ST*_ values detected across multiple years at several NC estuarine sites, including Hancock Creek (four of six comparisons), Clubfoot Creek (eight of 10 comparisons), and Slocum Creek (five of six comparisons). This inconsistent temporal structure likely reflects interannual variation in the amount and/or source of larval recruitment, driven by environmental variability in wind, currents and freshwater discharge during the pelagic larval stage [43,46,47]. The temporal genetic differences we observed among juvenile cohorts are consistent with sweepstakes reproductive success, in which environmental stochasticity causes a limited number of adults to disproportionately contribute to a given recruitment event. This generates chaotic genetic patchiness (CGP; [48]), or temporally unstable population genetic differentiation, even in the absence of persistent population subdivision. Similar patterns of temporally unstable genetic structure have been documented across numerous species with pelagic larval stages [6,49], suggesting that the dynamic recruitment patterns we observed in southern flounder reflect a broader pattern among highly dispersive coastal fishes. In addition to this temporal variability, we also observed substantial genetic variation among individuals within the same sampling locations. This suggests that estuarine nursery habitats may receive recruits from multiple spawning groups or source areas. Such mixed recruitment implies that individuals within a single location may originate from different parental populations or spawning events, further contributing to the subtle and temporally variable genetic patterns observed among sites. These findings highlight the importance of incorporating multi-year genetic data when evaluating population structure. For example, sampling only in 2014 would have yielded no significant pairwise *F*_ST_ values in North Carolina, while 2015 revealed significant differentiation at five of 15 comparisons. These conclusions differ substantially regarding the presence and distribution of fine-scale genetic structure. Therefore, conclusions drawn from a single sampling year represent only a snapshot of a dynamic system and could lead to uninformed management or broodstock sourcing decisions.

Previous studies found little evidence of population genetic structure within the Atlantic [12–14]. However, these studies were limited by lower marker resolution, reduced fine-scale sampling, and a lack of temporal replication. Earlier work using microsatellites or allozymes, as well as SNP based studies with fewer loci [14], may have lacked the power to detect weak or transient genetic structure. In addition, sampling across multiple years likely obscured temporal variation by averaging signals across cohorts. In contrast, our use of a larger genome-wide SNP dataset (~25,000 SNPs) with fine-scale spatial and temporal sampling was designed to overcome these limitations enabling detection of the subtle and dynamic structure we report here. The inconsistent fine-scale structure we observed within North Carolina likely reflects temporally variable recruitment and shifting spawning stock contributions rather than stable subdivision. As such, genetic structure at this scale is likely ephemeral and cohort-dependent, making it difficult to detect in studies that average across years or sample on broader spatial scales. These results suggest that the apparent lack of structure reported in earlier studies may reflect limitations in sampling design and marker resolution rather than true panmixia within Atlantic populations.

Within North Carolina, we detected several notable regional patterns. The southern New River region exhibited more consistent differentiation from other locations, potentially reflecting environmental influences on larval dispersal, recruitment, local selection, or spawning stock source. Honeycutt et al. [23] found higher temperatures in the New River region were associated with more male-biased sex ratios in southern flounder, likely due to a combination of genetic sex determination (GSD) and temperature-dependent sex determination (TSD), in which water temperature during early development can influence gonadal differentiation. This suggests that temperature-driven selection could be driving genetic differentiation in this region. Asynchronous reproduction may also be driving these differences. Honeycutt et al. [23] also reported that some New River region fish had similar growth patterns to fish sampled from South Carolina, where migration and spawning occur earlier [16], while others were smaller, suggesting a later spawning event. These observations indicate that some New River fish may be moving offshore to spawn earlier in the season than northern regions, leading to temporal spawning stock structure and genetic differentiation. Additional sampling from the New River region is needed to better understand how environmental conditions and reproductive timing shape genetic structure in this region. As ocean temperatures continue to rise across the southeastern Atlantic [50], further research with expanded sampling in this region will be important to determine how temperature-driven processes influence selection and reproductive behavior in southern flounder populations at local levels.

Albemarle Sound, the northernmost estuary in this study that we sampled in 2023, exhibited strong differentiation among sites both within and outside the Albemarle region. With only a single inlet connecting Albemarle to the broader Atlantic via Pamlico Sound, opportunities for gene flow may be constrained, leading to local differentiation even across small spatial scales within this Sound [51]. These findings raise the possibility that restricted oceanographic connectivity may contribute to the pronounced genetic structure observed in this region. To determine the mechanism causing increased genetic differentiation, and whether this is a consistent pattern in Albemarle Sound, will require additional sampling, including multi-year sampling.

## Conclusions

Our findings have direct implications for the management of southern flounder in both the Atlantic and Gulf of Mexico. The temporal variability in genetic composition we observed among recruiting juveniles, including significant interannual differentiation at multiple NC estuarine sites and at Bastrop Bayou in Texas, suggests that spawning stock contributions to estuarine recruitment shift among years. This has important implications for stock assessment, as genetic data averaged across years or drawn from a single cohort may not accurately reflect the diversity or composition of a spawning stock. Our findings are consistent with the prediction that maintaining large, diverse spawning stocks would buffer against the interannual variability we document, as larger stocks are more likely to include individuals spawning across a range of locations and times [52,53]. Incorporating multi-year genetic data into stock assessment frameworks will improve the ability to detect these dynamics, avoid bias toward single cohorts or recruitment events, and support management strategies that sustain both connectivity and adaptive potential [54].

Overall, our results demonstrate that southern flounder population structure is shaped by dynamic interactions among spatial, temporal, and environmental processes, leading to patterns that are often subtle and cohort dependent. Recognizing this variability is critical for accurately interpreting connectivity and recruitment across the species’ range. Our findings highlight the value of integrating fine-scale, multi-year genetic data to capture these dynamics and avoid misleading conclusions based on single-year or coarse-scale sampling. While our study focuses on southern flounder, the challenges of detecting fine-scale and temporally variable genetic structure are broadly relevant to other estuarine-dependent marine species with pelagic larval stages, particularly those experiencing population declines where accurate assessment of connectivity and spawning stock contributions is critical for management. The use of high-resolution genomic approaches, such as ddRAD sequencing, enables the analysis of thousands of SNP markers and further enhances the ability to detect variation in population structure. Moving forward, expanded spatial and temporal sampling, coupled with environmental data, will be essential for linking oceanographic processes, reproductive behavior, and genetic structure. Moreover, future work incorporating outlier locus analyses to identify signatures of selection across the genome would complement the neutral population structure described here and provide additional insight into local adaptation in this species. These efforts will strengthen management strategies that maintain population connectivity, preserve adaptive potential, and support the long-term resilience of this commercially important finfish species.

## Supporting information

S1 Table Number of samples analyzed post-filtering at each site and year. – indicates that the location was not sampled that year. Location # corresponds to maps in Figures 1 and 2.
(XLSX)

S2 Table Pairwise Fst values of NC and TX in 2014, 2015 and 2016. Highlighted values indicate significance based on 95% confidence intervals.
(XLSX)

S3 Table Population-level pairwise values of differentiation (F_ST_) for southern flounder collected from North Carolina Estuarine locations in 2014. Regions are specified in parentheses. Highlighted values indicate significance based on 95% confidence intervals.
(XLSX)

S4 Table Population-level pairwise values of differentiation (F_ST_) for southern flounder collected from North Carolina Estuarine locations in 2015. Regions are specified in parentheses. Highlighted values indicate significance based on 95% confidence intervals.
(XLSX)

S5 Table Population-level pairwise values of differentiation (F_ST_) for southern flounder collected from North Carolina Estuarine locations in 2016. Regions are specified in parentheses. Highlighted values indicate significance based on 95% confidence intervals.
(XLSX)

S6 Table Population-level pairwise values of differentiation (F_ST_) for southern flounder collected from North Carolina Estuarine locations in 2023. Regions are specified in parentheses. Highlighted values indicate significance based on 95% confidence intervals.
(XLSX)

S7 Table Pairwise Fst values for broad regions in NC and years. Significant values are highlighted.
(XLSX)

S8 Table Pairwise Fst values for each temporal location. Significant values are highlighted. Regions: PS, Pamlico Sound; NS, Neuse River; NR, New River; TX, Texas. A) Swanquarter (PS); B) Hancock Creek (NS); C) Slocum Creek (NS); D) Clubfoot Creek (NS); E) Mill Creek (NR); F) Virginia Creek (NR); G) Bastrop Bayou (TX).
(XLSX)
